# Changing Priorities in Vaccinology: Antibiotic Resistance Moving to the Top

**DOI:** 10.3389/fimmu.2018.01068

**Published:** 2018-05-30

**Authors:** Aldo Tagliabue, Rino Rappuoli

**Affiliations:** ^1^Institute for Genetic and Biomedical Research, CNR, Cagliari, Italy; ^2^GSK Vaccines, Siena, Italy

**Keywords:** antibiotic resistance, vaccination, reverse vaccinology, human immunology, public health

## Abstract

Antimicrobial resistance (AMR) is currently the most alarming issue for human health. AMR already causes 700,000 deaths/year. It is estimated that 10 million deaths due to AMR will occur every year after 2050. This equals the number of people dying of cancer every year in present times. International institutions such as G20, World Bank, World Health Organization (WHO), UN General Assembly, European Union, and the UK and USA governments are calling for new antibiotics. To underline this emergency, a list of antibiotic-resistant “priority pathogens” has been published by WHO. It contains 12 families of bacteria that represent the greatest danger for human health. Resistance to multiple antibiotics is particularly relevant for the Gram-negative bacteria present in the list. The ability of these bacteria to develop mechanisms to resist treatment could be transmitted with genetic material, allowing other bacteria to become drug resistant. Although the search for new antimicrobial drugs remains a top priority, the pipeline for new antibiotics is not promising, and alternative solutions are needed. A possible answer to AMR is vaccination. In fact, while antibiotic resistance emerges rapidly, vaccines can lead to a much longer lasting control of infections. New technologies, such as the high-throughput cloning of human B cells from convalescent or vaccinated people, allow for finding new protective antigens (Ags) that could not be identified with conventional technologies. Antibodies produced by convalescent B cell clones can be screened for their ability to bind, block, and kill bacteria, using novel high-throughput microscopy platforms that rapidly capture digital images, or by conventional technologies such as bactericidal, opsono-phagocytosis and FACS assays. Selected antibodies expressed by recombinant DNA techniques can be used for passive immunization in animal models and tested for protection. Antibodies providing the best protection can be employed to identify new Ags and then used for generating highly specific recombinant Fab fragments. Co-crystallization of Ags bound to Fab fragments will allow us to determine the structure and characteristics of new Ags. This structure-based Ag design will bring to a new generation of vaccines able to target previously elusive infections, thereby offering an effective solution to the problem of AMR.

## “Microbes Maketh Man”

Modifying the old saying “Manners maketh man” (reported by William Horman in *The Vulgaria* written in 1519), a few years ago the magazine The Economist published in its Leaders section a comment regarding the new vision of the interaction between microbes and man (1). The new saying clearly indicates that microbes have determined in many ways the evolution of the human species. At a first level, bacteria, viruses, fungi and archaea have literally become a part of us, forming the so called microbiota. A recent study has defined more precisely the number of bacteria present in our body, which is in the order of 39 trillion cells (2). Since the estimated number of human cells in the body (about 84% of which are red blood cells) is in the order of 30 trillion, the ratio between bacterial and human cells is about 1.3. The numbers may vary significantly from person to person and could change significantly with each defecation, ranging from 30 to 50 trillion in each individual. Women may also have a higher ratio of bacterial vs. human cells, because they have fewer red blood cells. This evaluation does not take into account fungi, viruses, and archaea, which all make up the human microbiota and would increase the ratio of microbes to human cells. Thus, we can consider ourselves like superorganisms, in which microbes do many jobs in exchange for the raw materials and the shelter their host provides. This alone shows how closely host and microbiota have co-evolved.

But microbes are also part of a living universe outside us, and often they act as parasites able to regulate the human life span. Over the entireness of the three million years of our species’ evolution, life expectancy has always been between 25 and 35 years until very recently, and infections have been the main regulators of our life span. By learning from observation of nature, human beings progressively improved their living conditions to the point that about 250 years ago life expectancy started to increase. In 1900, mankind had already reached a life expectancy of approximately 50 years (3). Nowadays, a child born in a high-income country can expect to live 85 years. The additional 35 years of life that we gained during the last century are substantially due to the conquest of infectious diseases, which used to kill 50% of people before the age of 20. These were viral diseases such as smallpox, rabies, measles, rubella, mumps, and bacterial infections such as diphtheria, tetanus, typhoid fever, and cholera (4). This result has been achieved primarily by improved hygiene, but also by treatment of infectious diseases with antibiotics and by their prevention throughout vaccination. As negative control of this important result, we have to consider the poor areas of our planet, where hygiene, vaccines, and antibiotics are not properly used even today. As a consequence, in these areas infections still represent a major cause of mortality, maintaining life expectancy below 50 years.

## Discovering a Great Tool Against Microbes

Antibiotics are an important example of how man can learn from nature to improve his own living conditions. The first observation that microbes are able to produces substances capable of killing pathogens came from the Italian scientist Vincenzio Tiberio in 1895, who showed the antibacterial activity of a natural substance produced by molds (5). Then Ernest Duchesne in France published in his doctorate thesis presented in 1897 (6) that the mold *Penicillium glaucum* possesses antibacterial properties. It was Alexander Flaming in 1928 that succeeded in definitely identifying the world’s first antibiotic. It was a substance isolated from the mold *Penicillium notatum*, defined as benzylpenicillin (penicillin G) (7). The first industrial production came however after Howard Florey and Ernst Boris Chain continued the work of Flaming in Oxford. Thus, after the Pearl Harbor attack in 1941 a mass production could be initiated. By 1944, enough penicillin was produced to treat the wounded soldiers in the Allied forces. The 1945 Nobel Prize in Physiology and Medicine was assigned to Flaming, Florey and Chain. The era of antibiotics had begun, and several other molecules produced by microbes followed penicillin. With antibiotics, mankind could claim an historical success in the eternal war against pathogens. However, it was soon clear that antibiotics were not the definitive weapon.

In a book published in 1975, Stanley Falkow wrote that “*we owe to chemotherapy (antibiotics) the debt of reducing the high mortality rate of many bacterial infections*” and to hygiene and vaccines the debt of preventing them, however “*in helping to solve some of the problems of infectious diseases, chemotherapy has created some problems of its own*” (8). The problem created by antibiotics was the generation of bacterial strains resistant to multiple antibiotics, an event reported for the first time in 1956, with the isolation in Japan of a strain of *Shigella flexneri* resistant to streptomycin, tetracycline, chloramphenicol, and sulfonamides.

Today, antimicrobial resistance (AMR) has grown out of proportion, and many pathogenic bacteria are resistant to multiple antibiotics, including *Neisseria gonorrhoeae, Staphylococcus aureus, Pseudomonas aeruginosa, Salmonella typhi, Shigella, Escherichia coli, Acinetobacter, Proteus, Klebsiella, Serratia, Streptococcus pneumoniae, Mycobacterium tuberculosis, Vibrio cholerae, Helicobacter pylori*, and others. In a few cases, bacteria became resistant to most of the available antibiotics and are on the verge of becoming untreatable. As a consequence, AMR is perhaps the most alarming emerging problem of infectious diseases. Globally, AMR already causes 700,000 deaths/year, and the forecast is that in 2050 it will cause 10 million deaths/year, higher than the 8.2 million deaths caused by cancer today. As an example we can look at *S. pneumoniae*, also known as pneumococcus, a human pathogen that is the major cause of community-acquired pneumonia, bacterial meningitis, bacteremia, and otitis media (9, 10). In the past, most strains of *S. pneumoniae* were sensitive to penicillin, whereas today penicillin resistance goes from 5 up to 60% in various parts of the world. Thus, with time old antibiotics become less effective or lose efficacy, making the search for new molecules with different mechanisms of action a priority (11).

Alarming documents, calling for action and asking for new antibiotics, have been issued by governments such as those of the UK and USA, by the European Union (EU), and by international organizations such as the World Health Organization (WHO), the United Nations General Assembly, and the World Bank and the G20. The interest in fighting the increase in AMR has intensified, and new incentives for research and development of new drugs have been deployed. In 2016, about 500 million US$ have been allocated to new and existing initiatives aiming to accelerate the development of new antibiotics^1^. For example, the Innovative Medicines Initiative (the biggest public–private program in biomedical science of the EU Commission) funded several projects defined as *New Drugs for Bad Bugs* ^2^. Other initiatives include CARB-X, a collaboration between US and UK partners^3^, and the Global Antibiotic Research and Development Partnership, a collaboration between the Drugs for Neglected Diseases *initiative* and the WHO^4^. It is also interesting a German proposal for a Global Union for Antibiotics Research and Development (GUARD), aimed at funding and coordinating a facility for antibiotics research and development.

On February 27th 2017, the WHO published a document^5^, which we partially report hereafter: “*This is the first ever list of antibiotic-resistant “priority pathogens”, a catalog of 12 families of bacteria that pose the greatest threat to human health. The list highlights in particular the threat of Gram-negative bacteria that are resistant to multiple antibiotics. These bacteria have built-in abilities to find new ways to resist treatment and can pass along genetic material that allows other bacteria to become multi-drug-resistant. The WHO list is divided into three categories according to the urgency of intervention, of critical, high and medium priority. The most critical group includes multidrug resistant bacteria that pose a particular threat in hospitals, nursing homes and among patients whose care requires devices (such as ventilators and blood catheters). The group encompass Acinetobacter, Pseudomonas and various Enterobacteriaceae (including Klebsiella, E. coli, Serratia, and Proteus). These bacteria can cause severe and often deadly infections such as bloodstream infections and pneumonia, and have become resistant to a large number of antibiotics, including carbapenems and third generation cephalosporins (the best available antibiotics for treating multi-drug resistant bacteria). The second and third tiers in the list—the high and medium priority categories— include other increasingly drug-resistant bacteria that cause more common diseases, such as Neisseria gonorrhoeae (the agent of gonorrhea) and Salmonella (causing food poisoning). This WHO action intends to promote initiatives of basic science and advanced R&D from both publicly funded agencies and the private sector, aiming to discover new antibiotics*.”

The WHO text continues as follows:

“Tuberculosis (TB) was not included in the list, although its resistance to traditional treatment has been growing in recent years, because TB is targeted by other dedicated programs. However, we must remember that TB now kills more people than any other pathogen (1.8 million in 2015), and it is therefore a most urgent priority. Other bacteria that are not included in WHO list, such as Group A and group B Streptococcus and Chlamydia, have low levels of resistance to existing treatments and do not currently pose a significant public health threat, but there is a risk that with time also these pathogens may become resistant. The list was developed in collaboration with the Division of Infectious Diseases at the University of Tübingen, Germany, using a multi-criteria decision analysis technique vetted by a group of international experts. The criteria for selecting pathogens in the list were: a) how deadly the infections they cause are; b) whether their treatment requires long hospital stays; c) how frequently they are resistant to existing antibiotics when people in communities catch them; d) how easily they are transmitted between animals, from animals to humans, and from person to person; e) whether they can be prevented (e.g., through good hygiene and vaccination); f) how many treatment options remain; and g) whether new antibiotics to treat them are already in the R&D pipeline.”

The WHO document strongly underlines the need for new treatments. Thus, the search for new antimicrobial drugs is and must remain a great priority. However, it is important to realize that the pipeline for new antibiotics is not very promising, thereby making unlikely that the problem will be solved along this line (12). On the other hand, another tool that, together with antibiotics, contributed to conquer and eliminate many infectious diseases, i.e., vaccines, have a very promising pipeline thanks to the new technologies (3). Thus, vaccines have the possibility to make a big contribution to the control of AMR.

## Resistance to Antibiotics and Vaccines

**Figure 1:**
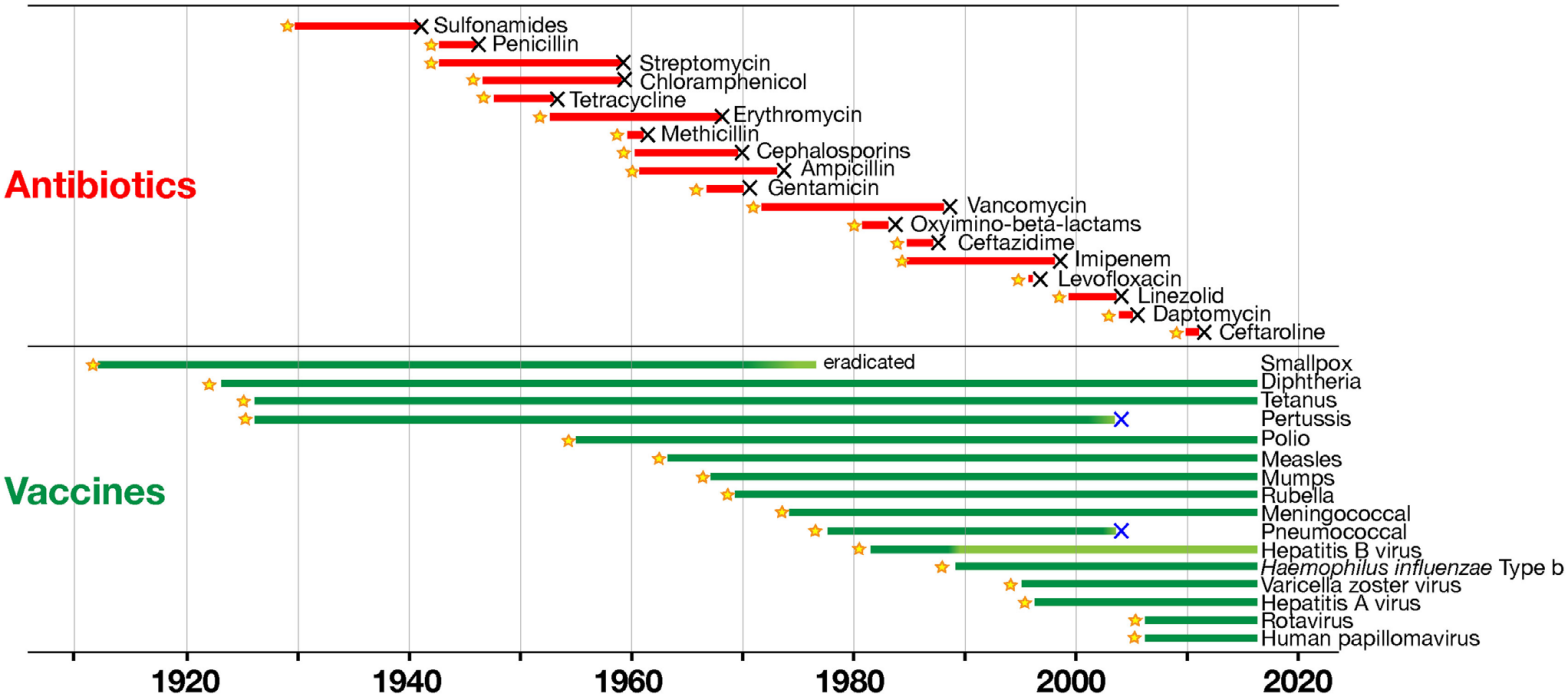
Time to detection of resistance of human pathogens to antimicrobials (in red) and to vaccines (in green). Black X symbols indicate insurgence of resistance, with lines starting at product introduction (yellow stars; except for smallpox vaccination that began much earlier; with modifications from Ref. (14) with the permission of the publisher).

The analysis of how vaccines and antibiotics contributed to conquering infectious diseases during the last century was originally published by the group of one of the authors of this paper (13), and more recently re-analyzed in depth by Kennedy and Read [(14), Figure 1]. This analysis shows that resistance to antibiotics inevitably emerges every time that a new antibiotic is introduced, starting a process of selection in the target bacteria that will eventually make that antibiotic useless. The consequence is that there is a continuous need of a fresh supply of novel antibiotics, to maintaining effectiveness of the therapeutic treatment. This strategy worked very well up to 1970s, when the identification of new antibiotics was abundant. However, since then the pipeline for new antibiotics has been drying out, and we have not been able to discover new classes of antibiotics (11). In marked contrast, Kennedy and Read show that we can use vaccines for a long time, generating no or very little resistance (14). Thus, vaccines can control infections over a long period of time without becoming obsolete. This occurs because vaccines work prophylactically and prevent the start of infections, while drugs work therapeutically on an ongoing infection in which bacteria proliferate and mutate, allowing the drug to select the resistant variants.

Furthermore, drugs are targeting few metabolic pathways on the pathogens, whereas vaccines induce a protective immune response against multiple antigenic targets. It can be concluded that selection has fewer opportunities to act upon vaccination than with antibiotic treatment. Still, both vaccines and antibiotics are very important in the control of infections. Table 1 reports the major differences in the mode of action of vaccines and antibiotics and provides information that can guide us to take advantage of their strengths and to minimize their weaknesses. From this comparison, it is evident that antibiotics are the only life-saving tool that we can use during acute bacterial infections, although their often improper or excessive use is causing bacterial resistance in a continuously increasing fashion. The availability of vaccines to control infections may allow us to decrease the use of antibiotics and to generate less AMR. This will permit a more efficient use of existing and new antibiotics during acute infections. As shown in Figure 1, there are, however, some cases in which resistance evolved after vaccination. This can be due to several causes, such as the fact that vaccines can protect from disease but may not have the capability to completely prevent pathogen colonization and transmission, as in the case of the acellular pertussis vaccine, or it can be caused by serotype replacement after vaccination with vaccines not including all serotypes, as in the case of the vaccines against *S. pneumoniae*. Thus, even for vaccines the search for better protective antigens (Ag) is very important, particularly for antibiotic-resistant infections.

**Table 1:** Comparison of the characteristics of vaccines and antibiotics in their capacity to fight pathogens.

Target/use	Vaccines	Antibiotics	Comments
Emergency use	No	+++	Antibiotics are immediately effective and are life saving during acute infections Vaccines require from 1 week to several months before they are fully protective
Memory (protection from diseases in the long term)	+++	No	Antibiotics are only effective while present in the body Vaccines induce a memory that last for many years
Eradication (of the infectious agent)	++	No	Vaccines allowed eradication of smallpox, and the elimination of polio, diphtheria, *Haemophilus influenzae*, meningococcus A and C, several strains of pneumococcus
Resistance (selection of resistant microbes)	+/−	+++	In nature, there are bacteria resistant to every antibiotic. Use, misuse, and abuse of antibiotics selects resistant bacteria and may generate superbugs that are resistant to most antibiotics There are very rare cases of resistance to vaccines.
Generation of new pathogens	−	++	Use, misuse, and abuse of antibiotics can select new pathogens as in the case of group B *Streptococcus*
Population use	++	−	Vaccines are most effective when used to vaccinate the entire population and generate herd immunity Antibiotics are most useful for the acute treatment of individual infections
Scientific progress (in the last 30 years)	+++	+/−	New powerful technologies such as glycoconjugation, genomics, structure-based antigen design, and adjuvants propelled the discovery and development of many novel vaccines Antibiotics did not benefit from the new technologies and during the last 30 years there was no discovery of new classes of antibiotics

In May 2016, a group of experts coordinated by the economist Jim O’Neill published a comprehensive report entitled “*Tackling drug-resistant infections globally*” (11). Among many indications to prevent the increasing global problem of AMR, an entire section was devoted to vaccines. Hereafter we report part of the section “*We must reduce the demand for antimicrobial so the current stock of drugs last longer*,” and in particular in the point Intervention 6 entitled “*Promote development and use of vaccines and alternatives*”:

“Vaccines can prevent infections and therefore decrease the demand for therapeutic treatments, reducing the use of antimicrobials thereby slowing the rise of drug resistance. Thus, vaccines should be eligible for the same incentives applied for antibiotic development. In particular, it is recommended 1) to use existing vaccines in humans and animals; 2) to renew impetus for early-stage research; 3) to sustain a viable market for vaccines.”

Similarly, the WHO document on priority pathogens stresses that the role of vaccines in the global AMR crisis remains of great importance.

## Promoting the Discussion About Vaccines as a Remedy to AMR

Since 2004, the year of 100th anniversary of the foundation of the Serology and Vaccinology Institute Achille Sclavo, a forum for the discussion of the most important issues of the vaccine world takes place every year in Siena, Italy. Each annual meeting aimed to analyze the state-of-the-art of important themes in the field of vaccines and expand the vision for the years to come. The participation of excellent speakers and expert discussants ensured the high quality of the meetings, whose conclusions were published in international journals (15–24). The meetings were organized close to a popular event, the horse race named “Palio di Siena,” for which the town is worldwide famous. Thus, those meetings were called the Palio Meetings. More recently, with the acquisition of the vaccine company in Siena by GSK, the venue of the meeting was moved to other locations in USA and Europe, but the traditional name was maintained. The topic of the meetings can vary but the mission is always based on one or more of the following pillars: (1) must define the state-of-the-art of cutting edge topics related to infectious diseases; (2) must advocate science policies to promote progress and improvement in human health; and (3) must be a strategic forum aimed to build new initiatives. Table 2 reports the topics of the Palio Meetings during the years.

**Table 2:** The Palio Meetings during the years.

Date	Meeting title	Location	Reference
2004 July 3	First International Congress on Emerging and Re-emerging Infections: Impact on Society, Economy and Medicine	Siena, Italy	
2005 August 17	Toward Global Health: Cooperation among Non-profit Organizations to Address Orphan Social Needs in Health: How to Build a Global Social Enterprise	Siena, Italy	
2006 August 17	Protagonists in Building Resources for Global Health and Delivering Health Tools to People who Most Need	Siena, Italy	
2007 July 3	Global Partnerships for Vaccination	Siena, Italy	(15)
2008 July 3	Meningococcus Scientific Exchange Meeting	Siena, Italy	(16)
2009 July 3	Rethinking Influenza: Can Planning Avoid the Panic?	Siena, Italy	(17)
2010 July 2	How Trust in Immunization Can be Built and Maintained	Siena, Italy	(18)
2011 July 2–3	Towards a Meningitis-Free World	Siena, Italy	(19)
2012 July 3	Prevention of Perinatal Group B *Streptococcus* Disease Through Maternal Immunization	Siena, Italy	(20)
2014 July 12	Enhancing Vaccine Immunity and Value	Siena, Italy	(21)
2015 July 18	Global Health 2015 – Mission Grand Convergence	Siena, Italy	(22)
2016 July 7	Emerging Infectious Diseases	Rockville, MD, USA	(23)
2017 July 6	Prioritizing Vaccines to Fight Microbial Infections	Wavre, Belgium	(24)

It was therefore almost mandatory that the subject of the 2017 Palio Meeting should be on the growing emergency caused by antibiotics failure, with the title “*Prioritizing vaccines to fight antimicrobial resistance*.” This event shortly followed a meeting organized by David Salisbury on 2017 March 29–30 at the Chatham House, in London (25). The London meeting provided a clear consensus that vaccines complement the actions of antibiotics and can contribute to control, reduce, and sometimes eliminate diseases caused by AMR pathogens, more than any other intervention. Thus, the main scope of the 2017 Palio Meeting was to build on the conclusions of the London meeting, and posed the question of how can we make vaccines achieve their full potential and become one of the top tools to tackle AMR. Indeed, the discussion led to conclude that there is a need to make stronger, more evidence-based cases supporting the importance of vaccines in AMR prevention (24).

One example is represented by vaccines against the main strains of *S. pneumoniae*, vaccines that have reduced pneumonia cases in the first decade of this century and in parallel have decreased the number of infections resistant to front-line antibiotics (26). The introduction in 2009 in South Africa of a pneumococcal vaccine achieved an analogous result. Furthermore, it is interesting to note that the high use of antibiotics, prescribed to treat opportunistic bacterial infections in people weakened by flu, is prevented when flu vaccines are employed. There is a need to make public the data generated by vaccine companies on vaccine effectiveness against AMR, and to continuously monitor the circulation of resistant bacterial strains. The discussion is continuing, and ideas and actions are becoming better defined (27). A global strategic effort to develop a portfolio of vaccines that target AMR is becoming mandatory.

## Evolution in Vaccine Research

Why vaccines are becoming an advantageous weapon to curb AMR? This is because their effectiveness in preventing infections has hugely improved, as a consequence of the enormous technological developments of the last two decades. Since the introduction of Jenner’s vaccine against smallpox in 1798, the field of vaccines has steadily progressed, but in the last years vaccine development has enormously benefited from the -omics approaches. Thus, new potential vaccine candidates can be discovered in much shorter time than in the past, when vaccines have been developed more empirically (3).

**Figure 2:**
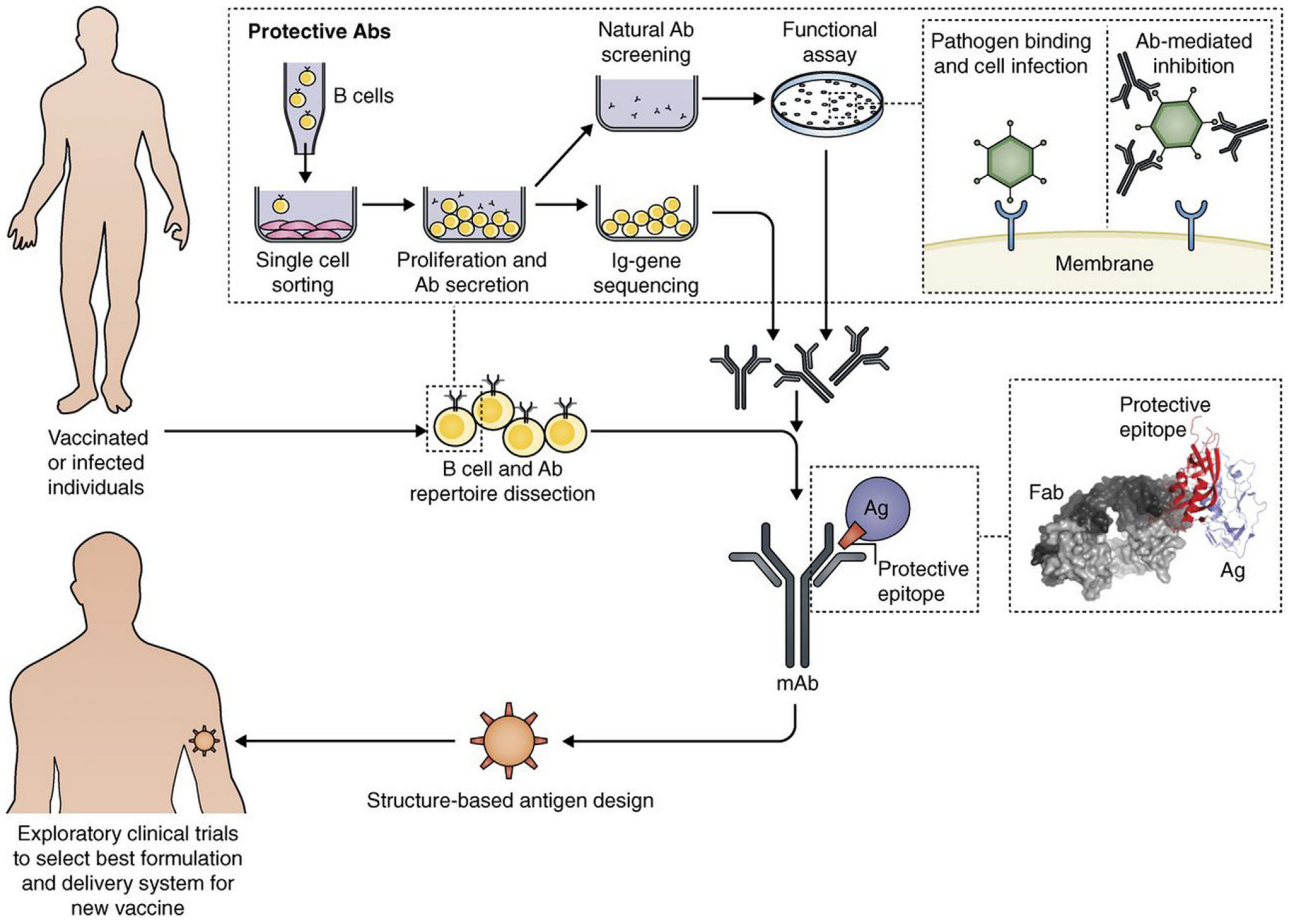
Interplay of B cell technology and structural biology in vaccine design, as shown with the Reverse Vaccinology 2.0 approach (from 35). Flow path representation of how the analysis of the human B cell repertoire leads to the identification of protective Abs from vaccinated or infected subjects. From upper left: Single B cell sorting and culturing enables a direct screening and selection of naturally produced Abs with desired functionality, and the recovery of the corresponding Ig gene sequences. This approach allows us to interrogate single-sorted B cells through direct screening of Ab functionality. From the recovered Ig sequences, we can produce the Abs of interest as recombinant proteins, and fine-tune their properties. The structural characterization of recombinant monoclonal Abs bound to their target antigen (Ag) leads to a detailed definition of the protective epitope. The right inset shows the co-crystal structure of an Ag–Ab (Fab) complex, identifying a protective epitope (red). Engineering of the protective epitope can lead to the design of a novel optimized immunogen. For example, we can mount the epitope in an oriented multi-copy array on a nanoparticle that will act as carrier and increase an epitope-focused immune response (“structure-based Ag design”). The new Ag can be developed with the best formulation or delivery system to then be tested in humans (from Ref. (30) with permission of the publisher).

The new techniques of genome sequencing introduced in the late 1990 completely changed the process for discovering novel vaccine Ags. The “*reverse vaccinology*” approach showed that, starting from sequence information, it is possible to discover the protective Ags without handling the microbes (28). A recently licensed vaccine against meningococcus B is the first vaccine produced with reverse vaccinology (29). During the last decade, vaccine design was further potentiated by new technologies, leading to an approach that has been named “*reverse vaccinology 2.0*” (30). As summarized in Figure 2, this approach takes advantage of human immunology for designing optimal vaccine Ags.

Thanks to better knowledge in handling human B cells and by selecting the most favorable donors, it is now possible to produce highly specific recombinant monoclonal antibodies (mAbs) and also their Ag-binding fragments (Fabs) (31). Further analysis by structural biology approaches brings to 3D studies of the target Ags complexed with the Fabs. It is also possible to discover the protective epitopes capable of inducing broadly neutralizing Abs (32–34). Furthermore, new computational approaches have allowed to obtain completely novel immunogens capable of inducing protection (35).

In viral infections, new structure-based powerful vaccine molecules have been already designed by screening human mAbs from convalescent people and obtaining the molecular structure of Ags and Ag–Ab complexes, as described earlier. Examples are the identification of the pentamer as key Ag for cytomegalovirus (CMV), and of the pre-fusion Ag of respiratory syncytial virus (RSV).

Until recently, the most promising CMV vaccine candidate was a recombinant form of the fusion protein gB. However, the human trial of gB combined with a potent adjuvant showed only moderate efficacy, and therefore the vaccine development was put on hold. Later, isolation of human mAbs from people previously exposed to CMV demonstrated that the most potent CMV neutralizing antibodies were not recognizing gB, but a complex Ag made by five proteins (pentamer). A recombinant form of the pentamer induced neutralizing antibodies that are orders of magnitude more potent than those induced by gB. The new Ag is a very promising candidate for a CMV vaccine and will soon undergo human trials (35).

In the case of RSV, it was possible to obtain a humanized mAb, palivizumab, that binds to an epitope present in the F protein in both the pre-fusion (pre-F) and post-fusion (post-F) conformation. Initial studies to develop an RSV vaccine were mainly focused on the use of the post-F protein that, unlike pre-F, is highly stable both as soluble Ag and when displayed onto virus-like particles. However, experimental vaccines based on whole virus, live attenuated virus, or post-F protein have failed to yield appropriate levels safety or efficacy. The scenario changed when the isolation and characterization of human neutralizing mAbs elicited by natural infection showed that the majority of antibodies are specific for the pre-F form of the protein and failed to cross-react with the post-F conformation. A structure-based design of a stabilized RSV pre-F protein was eventually obtained by complementing the crystal structure of the pre-F protein complexed with a highly neutralizing antibody with the neutralizing studies. The designed pre-F protein (DS-Cav1) could induce neutralizing antibodies 10–15 times more potent than those elicited by previous vaccines and is presently being tested in human trials (36, 37).

We can conclude that interrogation of human antibody responses can allow us to identify pathogen epitopes that are more likely to be protective and that are difficult to discover by conventional technologies. So far, isolation of human mAbs has been successfully used to identify viral Ags that could not be discovered by conventional technologies. On the other hand, there are no data yet regarding the identification of new Ags for antibacterial vaccines. After the proof-of-concept obtained with viral Ags, it would be very important to apply the same approach to the identification of novel bacterial Ags. This will allow us to design innovative vaccines against antibiotic-resistant pathogens, thereby effectively tackling the most pressing global health emergency.

## Conclusion

It is time to consider how to find an effective solution to fight antibiotic resistance, and win this battle in the never-ending war against pathogenic microorganisms. As discussed, the human species has experienced an impressive prolongation of its life expectancy and improvement in life conditions, due to hygiene, antibiotics, and vaccines. Now, one of these pillars has weakened to the point that it will affect some important medical methodologies, first of all important surgeries, but also immunosuppressive chemotherapy and consequently organ transplantation, a great success of the medicine of our era. Again, infectious diseases could severely reduce our life span, as we will not be able to survive important medical treatments or even accidental wounds. A possible solution in sight is that of developing a combined preventive-therapeutic approach, in which vaccines will be one of the two arms and chemotherapy the other one. There are reasons to believe that the combination of the two approaches will result in an overall success.

The main reason is the difference in the mode of action between vaccines and antibiotics. First of all, vaccines on their own are rarely capable to generate resistance. Another critical difference between antibiotics and vaccines is the rate of discovery of new effective molecules. In the past, new antibiotics were identified and regularly reached the clinic, particularly in the three decades after 1950. Since then, however, very few new molecules have been introduced in the clinical use. An opposite situation occurred in the case of vaccines, which have been developed at an increasing speed. As for today, 22 new vaccines became available since 1980. This is a consequence of the introduction of new technologies, such as recombinant DNA, that led to the generation of new synthetic sequences. Therefore, we have obtained a great reduction of the incidence of bacterial meningitis (caused by *Haemophilus influenzae, S. pneumoniae*, and *Neisseria meningitidis*) thanks to a new generation of very effective conjugated vaccines, generated by chemical technologies for covalently linking bacterial polysaccharides to proteins. More recently, genomic sequencing opened the access to a higher level in vaccine design, since it made possible to predict the thousands of proteins encoded by bacterial genes, and to identify those likely exposed on the cell surface, in search of new vaccine candidates. This approach, defined as “reverse vaccinology” has resulted in a first important protein vaccine against meningococcus B that is now in use worldwide (29). Finally, a better understanding of the mechanisms regulating the induction of a protective immune response has opened the possibility to introduce, in vaccine formulations, new moieties that can make them more effective. These substances are defined with the general name of adjuvants.

The difference in the mechanisms of action of vaccines and antibiotics is enormous. Antibiotics are families of molecules produced by microorganisms to kill microorganisms. What makes them effective is their capability to reach and poison targets across the strong barrier of the bacterial cell wall and avoid being ejected by potent efflux pumps. Any biochemical modification of the target microorganism can make the antibiotic inefficient. Among billions of bacteria present during an infection, such modifications can stochastically arise frequently. Vaccines are molecules with the capacity to evoke in the host a protective activity against infections. They do so by interacting with the immune system of the host, a system that during evolution has developed sophisticated mechanisms to recognize and destroy any kind of “danger” agents, essentially by distinguishing molecules that are different from self. The human immune system can potentially recognize any Ag in the universe, even those never encountered before, thanks to its complex gene rearrangement mechanisms. Thus, it is quite obvious that the potential of vaccines to protect us is extraordinary. And the more we learn about our immune system, the better we can design strategies and develop tools to protect our health from infections. For instance, in some cases it is now possible to cure established infections by administering the patient with specific antibodies produced in the lab with new technologies. In a way, these anti-infective antibodies could be considered as a new kind of antibiotic family, even though much more expensive.

For the time being, the strategy of combing antibiotics and vaccines remains the most sustainable option, which can allow us to avoid in an affordable way the AMR threat. We wish to stress again how serious this threat is, as we expect AMR will cause 10 million deaths/year from 2050.

Before embarking in complex combination studies, it is important to further investigate the role that existing vaccines could have against resistant infections. Indeed, there are some indications that an unconventional use of existing vaccines could provide important advantages. For instance, in New Zealand a new vaccine against meningitis B was introduced few years after the meningitis B outbreak of the end of the 1990. The vaccine was still produced with traditional techniques and was composed by bacterial outer membrane vesicles. Recent studies in the population vaccinated with this vaccine revealed protection against gonorrhea, a sexually transmitted infection induced by *N. gonorrhoeae* that is becoming resistant to antibiotics (38). The reason of this protection is likely due to the fact that bacteria causing meningitis and gonorrhea are genetically related. Furthermore, the current evidence shows that existing pneumococcal vaccines reduce AMR, due to the fact they prevent infection thereby reducing the carriage and transmission of antibiotic-resistant bacteria. Another example is the influenza vaccine that indirectly reduces the incidence of fever and sickness, thereby minimizing the use and, more often, the misuse of antibiotics.

The increasing AMR is one of the several alarming signals of the profound effects that human activities can have on our world and life on it. How can we try to solve the problem of infections that are resistant to antibiotics? The most critical point is the difficulty in obtaining new antibiotics. As already discussed, the classical approach does not work anymore, thus we need to devise a completely novel approach.

Passive immunization, i.e., the administration of immune antibodies, could perhaps be a solution, if we can solve the issue of sustainability. Nowadays, immune antibodies can be produced only in low amounts and with high costs. At the beginning of the last century, immune antibodies able to neutralize bacterial toxins were produced in big animals and largely employed, and contributed to building an industrial sector (“serum” institutes) that evolved in today’s vaccine industry. We hope that the new technologies will allow us to revive the serology concept and make it a new tool against infection.

A revolutionary approach would be to make the bacteria living within or on us, our microbiota, to become our allies in fighting the infections. As already mentioned, a large component of our body is bacteria (over 50%). An increasing number of studies indicate that gut microbiota is influencing our health and pathological conditions (39). Intestinal microbes can influence host energy metabolism (40), intestinal epithelial proliferation (41), and immune responses (42). It has been shown that the microbiota composition could influence vaginosis (43), obesity (44), inflammatory bowel disease (45), functional bowel disorders (46), allergies (47), and other diseases. An increasing number of studies suggest we can educate our microbiota to combat metabolic and chronic diseases. Could it be also the case in fighting infections?

Several candidate vaccines are in development pipelines since the last few years. For sure the emergency that we are facing will change the health priorities, and vaccines against antibiotic-resistant bacterial strains will move to the top.

## Author Contributions

AT wrote the manuscript. RR critically revised it.

## Conflict of Interest Statement

AT declares the lack of any commercial or financial relationships that could be construed as a potential conflict of interest. RR declares to be an employee of GSK Vaccines, Siena, Italy.

## Footnotes

^1^Boston Consulting Group, Federal Ministry of Health. *Breaking Through the Wall: a Call for Concerted Action on Antibiotics Research and Development* (2017). Available from: http://www.bcg.de/documents/file219507.pdf (Accessed: May 21, 2018).

^2^http://www.imi.europa.eu/content/nd4bb (Accessed: May 21, 2018).

^3^http://www.carb-x.org/about (Accessed: May 21, 2018).

^4^http://www.dndi.org/wp-content/uploads/2016/03/GARDP_Briefer_Document.pdf (Accessed: May 21, 2018).

^5^http://www.who.int/mediacentre/news/releases/2017/bacteria-antibiotics-needed/en/ (Accessed: May 21, 2018).
